# The Ethical Principles in Ethical Guidance Documents during the COVID-19 Pandemic in the United Kingdom and the Republic of Ireland: A Qualitative Review

**DOI:** 10.1017/S1049023X24000396

**Published:** 2024-10

**Authors:** Kesidha Raajakesary, Lucy Galvin, Kate Prendiville, Sarah Newport, Calum MacAnulty, Ghaiath Hussein

**Affiliations:** 1. Medical Student, School of Medicine, Trinity College, Dublin, Ireland; 2. Assistant Professor, Discipline of Medical Education, School of Medicine, Trinity College, Dublin, Ireland

**Keywords:** COVID-19, ethics, guidelines, pandemic

## Abstract

**Background:**

The sudden onset of the coronavirus disease 2019 (COVID-19) pandemic was accompanied by a myriad of ethical issues that prompted the issuing of various ethical guidance documents for health care professionals in clinical, research, and public health settings throughout the United Kingdom (UK) of Great Britain and Northern Ireland and the Republic of Ireland. The aim of this review was to identify the main principles in ethical guidance documents published in the UK and Ireland during the COVID-19 pandemic.

**Methods:**

This review used a qualitative review methodology with thematic synthesis to analyze the included ethics-related guidance documents, as defined in this review, published in the UK and Ireland from March 2020 through March 2022. The search included a general search in Google Scholar and a targeted search on the websites of the relevant professional bodies and public health authorities in the two countries. The ethical principles in these documents were analyzed using the constant comparative method (CCM).

**Results:**

Forty-four guidance documents met the inclusion and exclusion criteria. Ten main ethical principles were identified, namely: fairness, honesty, minimizing harm, proportionality, responsibility, autonomy, respect, informed decision making, duty of care, and reciprocity.

**Conclusion:**

The guidelines did not present the ethical principles in equal detail. Some principles lacked definitions, leaving them vulnerable to misinterpretation by the documents’ end users. Priority was frequently given to collectivist ethics over individualistic approaches. Further clarity is required in future ethical guidance documents to better guide health care professionals in similar situations.

## Background

Medical ethics has long been integral in decision making in health care, research, and policy setting during public health emergencies, especially those of a global scale. In total, the World Health Organization (WHO; Geneva, Switzerland) has declared seven Public Health Emergencies of International Concern (PHEIC) since 2000.^1^ Despite the number of recent PHEICs and the publication of numerous ethical guidance documents following previous pandemics and epidemics, neither the health care system nor the public were prepared for the difficulties the coronavirus disease 2019 (COVID-19) pandemic would bring.^2^

There has been a myriad of documents published advising health care professionals, researchers, and public health practitioners as to how they should make good decisions.^3–6^ The rapid development of the COVID-19 pandemic at the beginning of 2020 demonstrated an urgent need for ethical guidance specific to the public health emergency that was taking place.^7^ It introduced a new level of difficulty to everyday ethical decision making, as often decisions had a direct impact on multiple patients^8^ or affected large numbers of people.^9^ For example, the surge in numbers of patients needing ventilation and a limited number of machines meant that doctors needed to decide who should be prioritized and given life-saving treatment.^10^ Government departments and professional bodies such as medical councils across the United Kingdom (UK) and Ireland issued guidance, policies, and frameworks which presented advice and detailed instructions about important ethical considerations for decision making during the pandemic. For example, the Scottish Government published ethical guidance which discussed issues such as ethical allocation of scarce resources^11^ and the British Psychological Society (London, UK) issued guidance on conducting research with human participants during the COVID-19 pandemic.^12^ These documents were necessary as it soon became clear that health care systems, research, and everyday life would be profoundly impacted by this pandemic and that tough ethical decisions would frequently need to be made.^13^ These documents provided useful information; however, they were often difficult to access, extensive, and unclear.^14^

Due to this lack of clarity, it was evident that a review of the ethical guidance published in relation to the COVID-19 pandemic in the UK and Ireland was necessary. It was suspected that there were inconsistencies in the understanding and applications of the principles mentioned in these documents. Therefore, the aim of this review was to identify the ethical principles in ethical guidance documents and explore the consistency, or inconsistency, of the definition and application of these principles across guidance documents. It is hoped that potential issues and improvements are highlighted to aid public health professionals, clinicians, and researchers when making tough ethical decisions during future pandemics.

## Methods

### Search Strategy

A qualitative review using constant comparative method (CCM) analysis was used to analyze ethical guidance documents^15^ found through Google Scholar (Google Inc.; Mountain View, California USA) and the websites of relevant authorities. This method for comparison of documents was justified based on its efficacy, as in “Research in Disaster Settings: A Systematic Qualitative Review of Ethical Guidelines.”^16^ For this review, “ethical guidance documents” were defined as the publicly available documents that were systematically developed for the purpose of providing ethical guidance on the decisions that need to be made by the health care providers, researchers, and/or policymakers during the COVID-19 pandemic, and was published by:

1. Relevant health-related government departments and authorities and/or ministries of health; or
2. Health care professional bodies, including but not limited to clinical specialties’ societies, associations, and regulatory bodies, namely the Irish Medical Council (IMC; Dublin, Ireland) and the General Medical Council (GMC; London, UK) in Ireland and the UK, respectively; or
3. Any public health care authorities entitled to provide guidance on COVID-19-related issues of ethical concern.

This definition was needed as there were documents that were titled differently but were all relevant to the review question. For example, titles such as policy, principles, framework, and dimensions were all used, therefore the collective term “ethical guidance documents” was used for clarity. The relevant documents were those published from March 11, 2020 – when the WHO declared the COVID-19 pandemic^17^ – through March 2022. This range encompassed the timeline from the manifestation of COVID-19 to its deceleration in 2022.^18^ Inclusion and exclusion criteria are summarized in Table 1.

**Table 1.** Inclusion and Exclusion Criteria for this Review

Inclusion Criteria	Exclusion Criteria
Published in or translated into English	Not available in English
Published from March 11, 2020 through March 31, 22	Published before March 11, 2020 (before COVID-19 was declared a pandemic) or after March 31, 2022
Published in the UK or Ireland	Published outside the UK and Ireland
	Legal documents and documents dedicated to legal issues
	Documents with animal care guidelines

Abbreviations: UK, United Kingdom; COVID-19, coronavirus disease 2019.

As such, the search included three main steps that included two streams, described below. First, a search was conducted of the websites of all the relevant entities and bodies that fulfil the operational definition outlined above, followed by a search on Google Scholar following previous similar reviews,^16^ and then by a targeted search in the websites of the list of authorities and entities. The search was conducted using various combinations of the following keywords: “ethics,” “ethical,” “Ireland,” “Republic of Ireland,” “United Kingdom,” “UK,” “COVID-19,” “pandemic,” “Coronavirus 2019,” “framework,” “guid*,” and “policy.”

### Google Scholar

Although the documents targeted in this search are not strictly academic in nature and are unlikely to be published in peer-reviewed journals, an initial search on Google Scholar was done to serve two purposes. Firstly, to avoid missing any important guidance that could have been published in a peer-reviewed journal. Second, it was meant to help in identifying the main stakeholders who may be involved in the production of ethical guidance documents, which would help the targeted search, as described below. Throughout the search, there were two tracks: one for each of the included countries. All the results were pooled together and assessed by all reviewers in terms of fulfillment of inclusion criteria. In case of disagreement, the reviewers would discuss with the project supervisor (GH) until a consensus was reached for all the included/excluded studies.

Results were limited to the first two hundred hits “ordered by relevance in accordance with the methods used in numerous similar systematic reviews.”^9^ However, no results were eligible from this search as no ethical guidance documents were identified.

### Targeted Search

**Figure 1.**
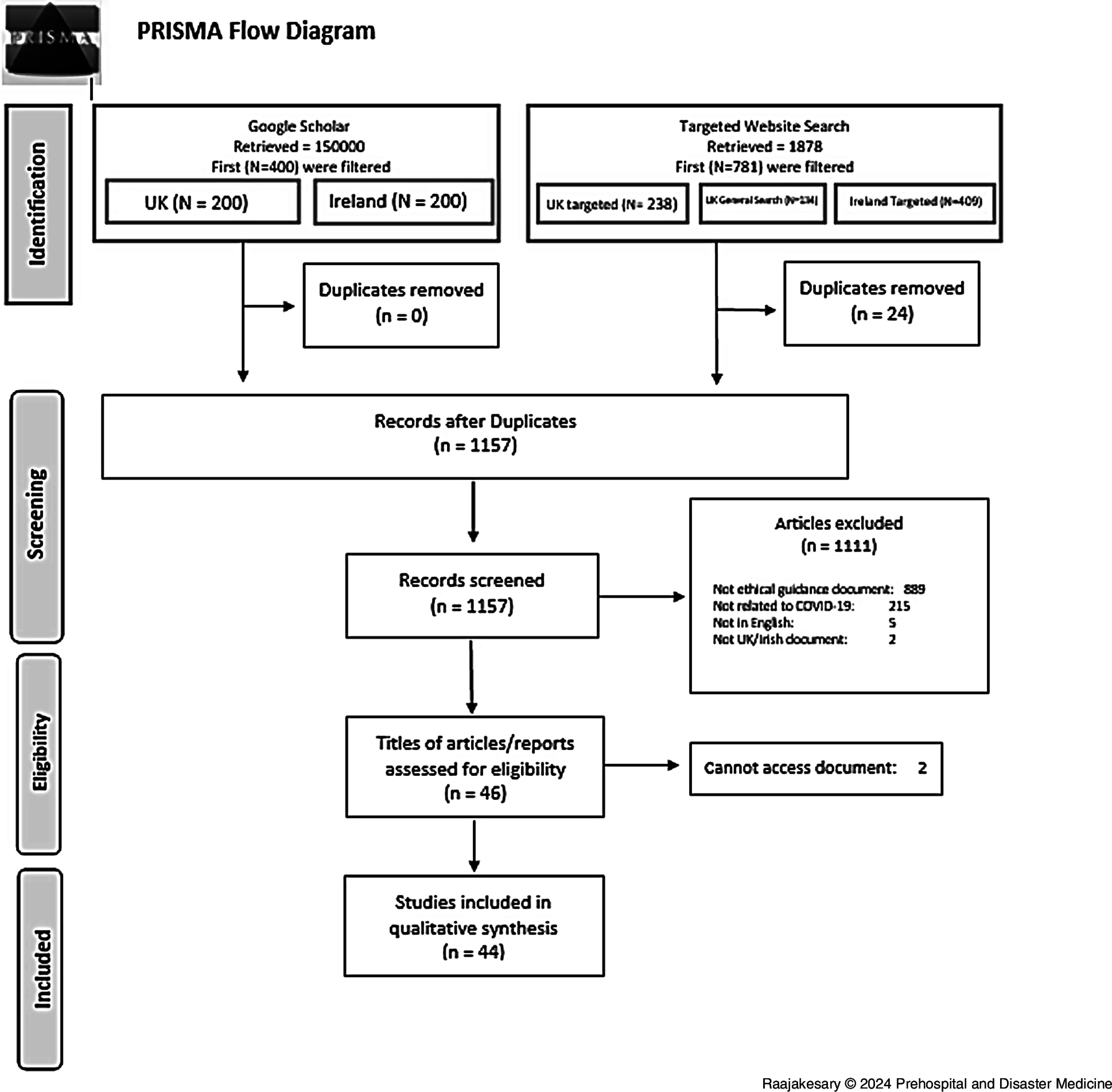
The PRISMA Diagram. Abbreviations: UK, United Kingdom; COVID-19, coronavirus disease 2019.

The next part of the search consisted of a targeted search that involved exploring the websites of specific authorities and bodies who were expected to have published ethical guidance related to COVID-19, for example the Health Service Executive (HSE; Dublin, Ireland) and the National Health Service (NHS; London, UK). The title and table of contents of the eligible documents were screened to decide on their inclusion. Forty-four documents were included in the review as shown in the PRISMA diagram (Figure 1).

## Results

Out of the screened documents (n = 1,157), 44 documents met the eligibility criteria, of which 16 were public health ethical guidance documents, 20 discussed clinical ethics, five focused on research ethics, two documents encompassed all three of these areas, and one paper discussed social work ethical guidance. Most documents were written by UK entities (n = 35/44), and the remaining were Republic of Ireland documents (n = 9/44). The publishing entities varied from departments of health to specific medical bodies, such as the British Medical Association (BMA; London, UK). Table 2 refers to the complete list of all included documents and their details such as publication date, document type, and principles identified in each document.

**Table 2.** Complete List of Documents Including Issuing Body, Date of Publication, Field, and Country of Publication

Doc. No.	Title	Issuing Body	Date of Publication	Field	Sub-Principles Discussed	Country
1	Acting Lawfully and Ethically Under the Coronavirus Act 2020	Southern Health NHS	April 13, 2020	Public Health	Respect, Fairness, Autonomy, Duty of Care, Proportionality, Reasonableness	UK
2	Brighton and Hove City Council COVID-19 Response	Brighton and Hove City Council	n/a	Public Health	Respect, Fairness, Responsibility, Minimizing Harm, Proportionality, Community	England
3	CILIP Policy Statement on COVID-19, Civil Liberties, and Professional Ethics	Chartered Institute of Library and Information Professionals (CILIP)	n/a	Public Health	Fairness, Autonomy, Responsibility, Community	UK
4	Coronavirus: Ethical Values and Principles for Health Care Delivery Framework	Welsh Government	April 12, 2020	Public Health	Respect, Fairness, Minimizing Harm, Proportionality, Community	Wales
5	COVID-19: A Practical and Ethical Guide to the Vaccination Scheme	Medical Protection UK	January 20, 2021	Public Health	Autonomy	UK
6	COVID-19 Guidance on Vaccination and Testing	Faculty of Occupational Medicine	January 22, 2021	Public Health	Autonomy, Minimizing Harm, Informed Decision Making	UK
7	COVID-19 Guidance: Ethical Advice and Support Framework	Department of Health NI	September 21, 2020	Public Health	Respect, Fairness, Autonomy, Honesty, Reciprocity, Responsibility, Minimizing Harm, Duty of Care, Informed Decision Making, Proportionality, Community	UK
8	Ethical Considerations in Responding to the COVID-19 Pandemic	Nuffield Council of Bioethics	March 17, 2020	Public Health	Respect, Fairness, Autonomy, Honesty, Responsibility, Proportionality, Community	UK
9	Guide to the Ethics of Surveillance and Quarantine for Novel Coronavirus	Nuffield Council of Bioethics	February 10, 2020	Public Health	Honesty	UK
10	Principles to Consider in Decision Making Resource Constrained vs Resource Unconstrained Decision Making	UK Clinical Ethics Network (UKCEN)	March 31, 2020	Public Health	Fairness, Duty of Care	UK
11	Putting Patients First during COVID-19: Our Ethical Decision-Making Process	Worcestershire Acute Hospitals	December 31, 2020	Public Health	Fairness, Autonomy, Honesty, Minimizing Harm, Duty of Care, Proportionality	UK
12	Receiving the COVID-19 Vaccine (Ethical Dimensions of COVID-19 for Frontline Staff - Appendix 1)	Royal College of Physicians	February 8, 2021	Public Health	Fairness, Honesty, Responsibility, Minimizing Harm, Duty of Care, Proportionality	UK
13	Responding to COVID-19 The Ethical Framework for Adult Social Care	Department of Health and Social Care	April 28, 2021	Public Health	Respect, Fairness, Responsibility, Minimizing Harm, Proportionality, Community	UK
14	RPS Guidance on Ethical, Professional Decision Making in the COVID-19 Pandemic	Royal Pharmaceutical Society (RPS)	April 8, 2020	Public Health	Respect, Fairness, Honesty, Reciprocity, Minimizing Harm, Proportionality, Community	UK
15	Ethical Framework for Decision Making in a Pandemic	Department of Health	September 24, 2020	Public Health, Clinical and Research	Respect, Fairness, Autonomy, Honesty, Reciprocity, Responsibility, Minimizing Harm, Duty of Care, Proportionality, Community	Ireland
16	Procedural Values for Decision Making in a Pandemic	Department of Health	September 24, 2020	Public Health, Clinical and Research	Fairness, Honesty, Reciprocity, Responsibility, Minimizing Harm, Duty of Care, Proportionality, Community	Ireland
17	Allocation Framework for Equitable Access to COVID-19 Vaccine(s)	Department of Health	December 16, 2020	Public Health	Fairness, Honesty, Responsibility, Minimizing Harm, Proportionality, Community	Ireland
18	COVID-19 Mandatory Vaccination-Ethical and Human Rights Considerations	Department of Health	February 17, 2022	Public Health	Fairness, Autonomy, Honesty, Reciprocity, Responsibility, Minimizing Harm, Duty of Care, Proportionality, Community	Ireland
19	Clinical Guidance for Managing COVID-19	Royal College of Nursing	February 22, 2022	Clinical	Fairness, Autonomy, Minimizing Harm, Informed Decision Making	UK
20	COVID-19: Ethical Guidelines for the Exit Strategy	Oxford Uehiro Centre for Practical Ethics, University of Oxford	October 1, 2020	Clinical	Fairness, Honesty, Minimizing Harm, Informed Decision Making, Proportionality, Community	England
21	COVID-19 – Ethical Issues: A Guidance Note	British Medical Association	n/a	Clinical	Respect, Fairness, Honesty, Reciprocity, Responsibility, Minimizing Harm, Duty of Care, Informed Decision Making, Proportionality, Community	England
22	COVID-19 Guidance: Ethical Advice and Support Framework	Scottish Government	April 3, 2020	Clinical	Respect, Fairness, Autonomy, Honesty, Reciprocity, Responsibility, Minimizing Harm, Duty of Care, Informed Decision Making, Proportionality, Community	Scotland
23	COVID-19 - Ethical Guidance in a Pandemic	Leeds Teaching Hospitals NHS Trust	May 24, 2021	Clinical	Respect, Fairness, Autonomy, Honesty, Reciprocity, Responsibility, Minimizing Harm, Duty of Care, Informed Decision Making, Proportionality, Community	UK
24	COVID-19: Ethical Considerations	Royal College of Psychiatrists	April 1, 2020	Clinical	Honesty, Reciprocity, Confidentiality, Duty of Care, Proportionality	UK
25	Devon-Wide Ethics Framework – Alignment to COVID-19 Response	Devon	May 28, 2020	Clinical	Respect, Fairness, Autonomy, Honesty, Reciprocity, Responsibility, Minimizing Harm, Duty of Care, Informed Decision Making, Proportionality, Community	UK
26	Ethical Dilemma Scenarios for Ambulance-Based Clinical Assessments during COVID-19 (Ethical Dimensions of COVID-19 for Frontline Staff - Appendix 2)	Royal College of Physicians	February 8, 2021	Clinical	Respect, Fairness, Autonomy, Honesty, Responsibility, Minimizing Harm, Duty of Care, Informed Decision Making, Proportionality	UK
27	Ethical Dimensions of COVID-19 for Frontline Staff	Royal College of Physicians	February 8, 2021	Clinical	Fairness, Honesty, Responsibility, Minimizing Harm, Duty of Care, Informed Decision Making, Proportionality, Community	UK
28	Ethical Framework for Utilization of Critical Care in Response to the COVID-19 Crisis	Government of Jersey	April 2020	Clinical	Fairness, Honesty, Responsibility, Minimizing Harm, Duty of Care, Informed Decision Making, Community	Jersey
29	Ethical Guidance on COVID-19 and Primary Care	Royal College of General Practitioners	November 17, 2021	Clinical	Respect, Fairness, Honesty, Responsibility, Minimizing Harm, Duty of Care, Proportionality, Community	UK
30	Ethical Guidance Published for Frontline Staff Dealing with Pandemic	Royal College of Physicians	February 8, 2021	Clinical	Respect, Duty of Care, Honesty	England
31	Ethics Framework for use in Acute Pediatric Settings during COVID-19 Pandemic	Royal College of Pediatrics and Child Health	January 8, 2021	Clinical	Fairness, Minimizing Harm, Proportionality	UK
32	Testing Times: An Ethical Framework and Practical Recommendations for COVID-19 Testing for NHS Workers	Healthcare Improvement Studies Institute	June 2020	Clinical	Fairness, Autonomy, Honesty, Responsibility, Proportionality	UK
33	COVID-19: Ethical Issues when Demand for Life-Saving Treatment is at Capacity	British Medical Association	n/a	Clinical	Respect, Fairness, Autonomy, Honesty, Reciprocity, Responsibility, Minimizing Harm, Duty of Care, Informed Decision Making, Proportionality, Community	England
34	Ethical Considerations Relating to Critical Care in the Context of COVID-19	Department of Health	September 24, 2020	Clinical	Fairness, Honesty, Minimizing Harm, Duty of Care	Ireland
35	Ethical Considerations Relating to Long-Term Residential care Facilities	Department of Health	September 24, 2020	Clinical	Respect, Fairness, Autonomy, Honesty, Reciprocity, Responsibility, Minimizing Harm, Duty of Care, Proportionality, Community	Ireland
36	Ethical Considerations for PPE use by Health Care Workers in a Pandemic	Department of Health	September 24, 2020	Clinical	Reciprocity, Minimizing Harm, Duty of Care, Proportionality	Ireland
37	HSE Guidance Regarding Cardiopulmonary Resuscitation and DNAR Decision Making during the COVID-19 Pandemic	National Quality Improvement Team	August 30, 2020	Clinical	Autonomy, Honesty, Responsibility, Minimizing Harm, Proportionality	Ireland
38	Interim Rights-Based Guidance on Implementing Infection Prevention Control Measures and Mitigating Risk in Disability Services	Health Service Executive	October 19, 2020	Clinical	Fairness, Autonomy, Honesty, Responsibility, Minimizing Harm, Duty of Care, Proportionality	Ireland
39	COVID-19 Pandemic - Ethical Guidance for Social Workers	British Association of Social Workers	April 1, 2020	Social Work	Respect, Fairness, Honesty, Minimizing Harm, Duty of Care, Informed Decision Making, Proportionality, Community	England
40	Ethical Guidelines – Collecting COVID-19	Science Museum	April 1, 2020	Research	Respect, Honesty, Responsibility, Minimizing Harm, Proportionality, Community	UK
41	Ethics Best Practice Guidance on Conducting Research with Human Participants during COVID-19	British Psychological Society	August 18, 2020	Research	Respect, Autonomy, Minimizing Harm, Proportionality	England
42	Guidance for UCL Researchers Regarding On-Going Research and Ethical Approval in Light of the COVID-19 Pandemic	University College London (UCL)	March 27, 2020	Research	Autonomy, Honesty, Proportionality	England
43	Research Ethics and Governance Guidance for Restarting Research Activities which have been Paused due to COVID-19	University of Birmingham	n/a	Research	Minimizing Harm	England
44	Research Ethics Guidance during COVID-19 - Glasgow	Glasgow Caledonian University	n/a	Research	Reciprocity, Responsibility, Minimizing Harm	UK

Abbreviations: NHS, National Health Service; UK, United Kingdom; COVID-19, coronavirus disease 2019; PPE, personal protective equipment; HSE, Health Service Executive.

This review identified 10 principles (Table 3).

**Table 3.** Number of Documents that Mentioned Each Ethical Principle

Ethical Principle	Number of Documents
Fairness	37
Honesty	30
Minimizing Harm	28
Proportionality	27
Responsibility	25
Autonomy	20
Respect	20
Informed Decision Making	19
Duty of Care	13
Reciprocity	12

## Discussion

The COVID-19 pandemic has raised numerous ethical questions, such as the debate surrounding the allocation of hospital beds or the fair distribution of vaccines. These unprecedented times caused various groups of experts or entities to publish ethical guidance documents that were focused on dealing with COVID-19. A previous study called for clarity, consistency, and fairness when it comes to national ethical guidance in relation to COVID-19.^7^ There are now several ethical guidance documents to guide health care professionals in their decision-making processes during the pandemic, as demonstrated in these results. This review focused on the contents of those documents, what the main principles were, and how they were discussed.

### Fairness

Prior to the COVID-19 pandemic, the UK Government published an ethical framework for pandemic influenza, which emphasized the importance of fairness – “Everyone matters equally. People with an equal chance of benefiting from a resource should have an equal chance of receiving it – although it is not unfair to ask people to wait if they could get the same benefit later.”^6^ This framework was referenced in several British COVID-19 ethical guidance documents, and its definition of fairness was repeated in four of the included documents.^6,8,19,20^ The Irish Department of Health’s description of fairness echoed that of the UK government closely, stating additionally that: “A fair decision is also one that might treat some people differently but for clinically sound reasons.”^8^ It is fair to ask patients who will benefit equally later to wait when resources are scarce.^19,20^ Guidance documents explained that providing fair care to patients must show “respect for them as individuals - of equal moral worth with all others.”^8,21^

### Honesty

Honesty and transparency were paramount in decision making during the pandemic. As the Scottish government^11^ outlined: “Decision-making processes should be fair and equitable, as well as transparent. Decision makers need to be honest with patients and the public about how decisions are made, in a way that they are able to understand.” Similarly, the BMA discussed this: “Open and transparent decision making: good decisions will be as inclusive, transparent, and reasonable as possible. They should be rational, evidence-based, the result of a reasonable process, and practical in the circumstances.”^22^ However, only a few discussed honesty outside of decision making. For example, the University of Oxford (Oxford, England) mentioned transparency in disseminating information: “The Government should be as open and transparent as possible about the risks of the virus, the costs of the lockdown so far, and the estimated costs of any future lockdown, as well as alternative strategies.”^23^

### Minimizing Harm

Nonmaleficence is the obligation of health care professionals not to harm patients (ie, to minimize harm).^3^ This is one of the foundational principles of health care ethics; therefore, it is not surprising that this was a recurrent theme throughout almost all of the documents. Minimizing harm was discussed from individualistic and collectivist perspectives. One document stated that harm should be minimized individually using evidence-based decisions to consider where medical interventions are appropriate, and societally by ensuring treatments are most available for those most likely to benefit from them.^24^ The NHS Constitution also states: “We accept that some people need more help, that difficult decisions have to be taken – and that when we waste resources, we waste opportunities for others.”^25^ Even prior to the pandemic, the same approach was advised; however during the pandemic, decisions of this nature had to be made more frequently than ever. There was no clear definition of what it means to “minimize harm,” however there were references to utilitarianism and individualism as the two major frameworks for minimizing harm.

Flexibility and responsiveness were highlighted in the context of minimizing harm: “Plans must be adaptable to changing circumstances”^22^ and “As the clinical situation evolves, both at the individual and population level, decisions require regular review with clear guidance at both national and regional level.”^26^ It could be argued that all guidance found throughout these documents was ultimately aimed at minimizing harm. For that reason, it is unsurprising that it featured in the majority of documents.

### Proportionality

The concept of proportionality in decision making appeared throughout the literature and encompassed several sub-themes, including fair allocation of resources and being consistent and practical. Proportionality and its sub-themes are interlinked with informed decision making. It is possible that most documents referred to proportionality because of the increased demand for finite resources, which was evident during the pandemic. The health care system was forced to make decisions with suboptimal resources and actions taken could not be ideal in all cases. Proportionate responses were defined by decision makers using “the most accurate information they can” and acting “flexibly and in proportion to the risks and benefits of individuals.”^26^ For example, Northern Irish guidance said that during the pandemic, decisions which balance risk and reward of treatment, such as cancer treatments, must be taken on a “case by case basis.”^26^ The BMA emphasized the likelihood that doctors will have to make decisions about a person’s eligibility for treatment based on their “capacity to benefit quickly” and highlighted that there cannot be a simple “cut off” policy with regard to age or disability.^22^

Particular attention was drawn in some cases in the literature to more pandemic-relevant issues, such as the ethics surrounding withdrawal or refusal of treatment to allow resources to be redistributed to those who will benefit more. On this, the BMA commented that: “To refuse someone potentially life-saving treatment where someone else has a higher priority for the available treatment” is “both lawful and ethical” where appropriate prioritization policies are followed.^22^ Once again, collectivist ethics were emphasized, while individual freedom was of lesser importance. However, this is not to say that all guidance completely ignored individual rights. The Irish Department of Health discussed the principles of “least infringement” and “least restrictive alternative” in the context of proportionality, and necessity when considering policies related to public health. They explained that: “The policy that entails the least intrusion on personal rights and freedoms, capable of achieving the public health goal, should be implemented.”^8^ Personal rights should be respected as much as possible, but it is imperative this does not come at the expense of public health. Treatment must be appropriate to need, but in the circumstances the pandemic presents, the needs of patients far outweigh the health care system’s capacity.

### Responsibility

In many situations arising in the pandemic, including allocation of scarce resources and vaccines, it was important that the principle of responsibility was at the forefront of decision making.^8,26^ The responsibilities of individuals during the pandemic forced them to make sacrifices. A shift towards collectivist ethics in a variety of scenarios was evident in several documents.^24,27^

The meaning and roles of “responsibility” have been considered in detail.^28^ Responsibility “has a control, and perhaps an epistemic, condition” and individual, personal, and moral responsibility are all mentioned, in addition to unqualified responsibility. It is also noted that the responsibility a doctor has to patients could be called the “obligation-sense of responsibility,” as opposed to the “accountability-sense of responsibility” in which a person has control over behaviors but fails to behave appropriately and thus may be held accountable.^28^ In the guidance documents explored in this qualitative review, the term “accountability” was given more weight than “responsibility,” having several clear definitions and directions.

Responsibility was perhaps most clearly described in the context of research. Scientific integrity was a major aspect of responsibility, thus research during the pandemic should be conducted in a manner which “ensures its quality, integrity, and contribution to the development of knowledge and understanding.”^12^

### Autonomy

Autonomy was defined as: “The ability of an individual to direct how he or she lives on a day-to-day basis according to personal values, beliefs, and preferences. In health and social care, this involves the person using our services making informed decisions about the support that he or she receives.”^29^ The requirement for people’s autonomy presented significant public health challenges during the COVID-19 pandemic, therefore it was concerning that this principle did not feature in several guidance documents. Although all Irish documents alluded in some capacity to respecting individuals’ wishes, six of the nine did not explicitly mention the term “autonomy.” However, issues relating to autonomy in pandemic-specific scenarios were well-highlighted, particularly in UK documents. The limitations created by patient isolation and personal protective equipment (PPE) meant extra effort was required to ensure that discussions regarding capacity and consent took place with patients and their families in a timely and sensitive manner.^26^ Testing for COVID-19 had to consider the principles of informed choice and autonomy, and individuals could decline tests for a range of reasons.^30^ Notably, Ireland did not provide ethical guidance in relation to COVID-19 testing, apart from in a guidance document specifically for those with disabilities^31^ and in guidance for long-term residential care facilities.^32^ This leaves a large proportion of the Irish population for whom there is no clear guidance in relation to their choice, or lack thereof, in testing for COVID-19.

### Respect

Generally, respect in medicine is defined as respect for persons, including respect for the autonomy of a patient and their right to self-determination.^33^ It is an integral pillar of health care and therefore dictated conversations in ethical decision making during the pandemic. The documents that failed to mention respect were vaccine allocation and research guidance documents that focused more on the principles of fairness, informed consent, and honesty and transparency.^20,34^ The Northern Irish guidance framework described the principle of respect to be a minimum that is expected of all health care professionals.^26^ The documents did not describe what respect or dignity was, but instead there was an implication that health care decision makers would already be aware of the importance of respect and dignity as it was key to health care in general. Furthermore, dignity was inherently linked to respect. Dignity was mentioned as a “fundamental” of care, especially when it comes to end-of-life care.^11^

### Informed Decision Making

Informed decision making involves decisions which are lawful, based on the best available evidence, and based on established guidelines.^27^ The Scottish government’s guidance acknowledged the additional pressures on the health care system, but emphasized that: “Clinicians must continue to act within the law.”^11^ This ensures patient safety as well as protecting clinicians from possible litigation. The BMA’s guidance states all decisions on resource allocation must be both “based on the best available clinical data and opinion” and “agreed on in advance where practicable, while recognizing that decisions may need to be rapidly revised in changing circumstances.”^22^ The Oxford Uehiro Centre (Oxford, England) highlighted the importance of the public understanding the evidence on which governments base decisions.^23^ With the increasing prevalence of misinformation, clarity on what makes evidence the “best” would help to eliminate misconceptions and protect both clinicians and patients.

### Duty of Care

Duty of care is: “Inherent to all codes of ethics and professional standards for health care professionals,”^8^ and it could be considered as the “obligation-sense of responsibility,”^28^ as mentioned above. Many documents mentioned that duty of care is the duty to maximize benefit and minimize harm, but there it was not clearly defined in any of the documents. Closely linked to duty of care is maximizing benefit. As with minimizing harm, there was a distinction between “the desire to maximize the benefits to the population” and the “duty to care for each individual,”^26^ making it difficult to decide on one simple definition for “duty of care.” A utilitarian approach was recommended by Leeds Teaching Hospital (Leeds, UK) and the BMA,^22,24^ which maximizes public benefit rather than individual benefit.

### Reciprocity

Reciprocity “requires that society supports those who face a disproportionate burden in protecting the public good and takes steps to minimize the risks and burdens as far as possible.”^8^ Ethical guidance documents highlighted a moral obligation to protect workers from harm and minimize the increased risks to frontline staff in the COVID-19 pandemic.^26^ Reciprocity was outlined as a way of “showing support and solidarity”^8^ for health care workers. The increased burden and “moral distress”^26^ imposed by the pandemic highlighted the importance of “proportionate and effective physical, moral, psychological, and pastoral support”^26^ for health care workers. Just twelve of the forty-four documents mentioned reciprocity as a principle to be considered when making decisions during the COVID-19 pandemic. The burnout experienced by health care workers during the pandemic highlights the need to include reciprocity more widely in future ethical guidance documents.

The four pillars of medical ethics, autonomy, beneficence, nonmaleficence, and justice, have long been used by health care professionals for making ethical decisions.^3^ These principles were prevalent throughout the documents. However, the themes covered in the documents exceed those four main principles. This seems to be a result of the additional ethical issues raised during the pandemic. An increase in the volume of ethical challenges called for further ethical guidance which extended beyond the guidance given for “normal” circumstances.

There was variation between the documents, but some key principles were prevalent throughout the majority. These principles include honesty, fairness, proportionality, informed decision making, duty of care, and minimizing harm. In contrast to that, autonomy, responsibility, proportionality, reciprocity, and respect appeared less frequently. Although these are important principles, perhaps the professional entities felt that they were less of a priority in the context of the COVID-19 pandemic.

The various principles were discussed at length in the documents. However, there was a lack of definitions of the principles themselves - respect, minimizing harm, duty of care, reciprocity, and informed decision making were not clearly defined in any of the included documents. It is assumed that the reader is already familiar with these ethical principles; however, this leaves them vulnerable to misinterpretation. Definitions could be useful in these documents, especially because many new doctors and nurses were “thrown in the deep end” without adequate training due to the staff shortages. This inadequate training also sheds light on the lack of ethics pertaining to staff training within the literature.

## Limitations

The scope of this review was narrowed to UK and Irish documents only, to account for the large number of documents that would be reviewed. This, however, limits the review’s generalizability pertaining to global ethics. Ideally, the review would study all ethical guidance documents published since the COVID-19 pandemic began; however, this is unrealistic due to time constraints and limited resources – this review was conducted part-time by medical students.

Secondly, this review was limited to guidance documents published in the English language only. Further research into European health care ethical guidelines published in other languages could enhance this review as Ireland often heeded advice from the European Medicines Agency (Amsterdam, Netherlands) regarding vaccine allocation and approval.^34^ The UK used its own vaccine approval agency, and the European documents could have been added as another layer of research to compare ethical guidance documents in more regions. In addition, the influence of the WHO’s guidance during the pandemic was not included in this review.

Thirdly, this review only included guidance documents published by governmental agencies or professional bodies. Had the scope been widened to include legal documents, the review could have explored the unique legal ethical challenges pertaining to the COVID-19 pandemic. This pandemic is the first of its type in over a century whereby the world came to a standstill for months as the world was put under “lockdown.” This has triggered several cases challenging the ethics behind the enforcement of these lockdowns.^35,36^ Legal documents could have aided the public health ethics aspect of this review and may have showcased further ethical principles relevant to this pandemic.

## Conclusions

This systematic qualitative review gives an overview of the main ethical principles in ethical guidance documents published by governments and professional bodies in the UK and the Republic of Ireland during the COVID-19 pandemic. Overall, ten key principles were found to be most referenced in the literature; however, some appeared more frequently than others. The review found, however, that the principles of reciprocity, honesty and transparency, and autonomy were not mentioned or referred to as often as other principles. This raises the question of whether the health care authorities issuing advice placed more importance on certain principles and, if so, why that might be. If there was no reason for the exclusion of some principles in papers, it is evident that the guidance documents need to be reviewed to ensure that all ethical principles relevant to decision making during the COVID-19 pandemic are included.

Going forward, it is recommended that guidance documents give definitions for the included ethical principles to ensure clear and consistent dissemination of knowledge. For future studies, it would be worth exploring how these principles were implemented within health care systems and their impact on providing ethical health care. In addition, this review explored the principles used by UK and Irish bodies. It may be worth exploring similar studies from other countries to gain a global perspective for ethical guidance.
