# The Ethical Principles in Ethical Guidance Documents during the COVID-19 Pandemic in the United Kingdom and the Republic of Ireland: A Qualitative Review – CORRIGENDUM

**DOI:** 10.1017/S1049023X24000694

**Published:** 2024-10

**Authors:** Kesidha Raajakesary, Lucy Galvin, Kate Prendiville, Sarah Newport, Calum MacAnulty, Ghaiath Hussein

The above-mentioned article was originally published with multiple references to a “Qualitative Systematic Review,” including in the article title. These instances have since been updated to simply “Qualitative Review.”

The journal apologizes for this error.
